# Maternal High-Fat Diet During Pre-Conception and Gestation Predisposes Adult Female Offspring to Metabolic Dysfunction in Mice

**DOI:** 10.3389/fendo.2021.780300

**Published:** 2022-01-17

**Authors:** Brian Akhaphong, Brigid Gregg, Doga Kumusoglu, Seokwon Jo, Kanakadurga Singer, Joshua Scheys, Jennifer DelProposto, Carey Lumeng, Ernesto Bernal-Mizrachi, Emilyn U. Alejandro

**Affiliations:** ^1^ Department of Integrative Biology & Physiology, University of Minnesota, Minneapolis, MN, United States; ^2^ Department of Pediatrics, Division of Diabetes, Endocrinology, and Metabolism, University of Michigan Medical School, Ann Arbor, MI, United States; ^3^ Department of Internal Medicine, Division of Metabolism, Endocrinology and Diabetes, Brehm Center for Diabetes Research, Ann Arbor, United States; ^4^ Diabetes, VA Ann Arbor Healthcare System, Ann Arbor, MI, United States; ^5^ Miami VA Healthcare System and Division Endocrinology, Metabolism and Diabetes, University of Miami, Miami, FL, United States

**Keywords:** maternal obesity, inflammation, diabetes, fetal programming, high-fat diet, Western-diet, metabolic dysfunction, sex dimorphism

## Abstract

The risk of obesity in adulthood is subject to programming in the womb. Maternal obesity contributes to programming of obesity and metabolic disease risk in the adult offspring. With the increasing prevalence of obesity in women of reproductive age there is a need to understand the ramifications of maternal high-fat diet (HFD) during pregnancy on offspring’s metabolic heath trajectory. In the present study, we determined the long-term metabolic outcomes on adult male and female offspring of dams fed with HFD during pregnancy. C57BL/6J dams were fed either Ctrl or 60% Kcal HFD for 4 weeks before and throughout pregnancy, and we tested glucose homeostasis in the adult offspring. Both Ctrl and HFD-dams displayed increased weight during pregnancy, but HFD-dams gained more weight than Ctrl-dams. Litter size and offspring birthweight were not different between HFD-dams or Ctrl-dams. A significant reduction in random blood glucose was evident in newborns from HFD-dams compared to Ctrl-dams. Islet morphology and alpha-cell fraction were normal but a reduction in beta-cell fraction was observed in newborns from HFD-dams compared to Ctrl-dams. During adulthood, male offspring of HFD-dams displayed comparable glucose tolerance under normal chow. Male offspring re-challenged with HFD displayed glucose intolerance transiently. Adult female offspring of HFD-dams demonstrated normal glucose tolerance but displayed increased insulin resistance relative to controls under normal chow diet. Moreover, adult female offspring of HFD-dams displayed increased insulin secretion in response to high-glucose treatment, but beta-cell mass were comparable between groups. Together, these data show that maternal HFD at pre-conception and during gestation predisposes the female offspring to insulin resistance in adulthood.

## Introduction

The Barker hypothesis described that there are critical and sensitive time points during development and different timing of exposure *in utero* could lead to altered phenotypes that can impact the metabolic health trajectory of the offspring in adulthood (1). Suboptimal intrauterine environment during these critical developmental periods, such as over or under nutrition can impact postnatal health outcomes or modulate risk of metabolic diseases such as Type 2 diabetes (T2D) in the adult offspring (2).

Studies demonstrate that different timing of exposure *in utero* could lead to altered phenotypes in the offspring depending on what cells and tissues are developing at that time of insult. For example, we and others have shown that pancreatic beta-cells are very sensitive to nutrient changes *in utero*, such that maternal low-protein diet throughout pregnancy reduces beta-cell mass at birth and impairs glucose homeostasis of the adult offspring. Maternal low-protein diet during pregnancy (starting at embryonic day 0.5, LP0.5) reduces beta-cell mass of offspring at birth and impairs insulin secretion in adulthood (3). However, maternal low-protein diet during the last week of pregnancy did not alter beta-cell mass of offspring at birth (4). Effects of undernutrition during gestation in the Dutch Famine cohort had small-for-gestational age (SGA) weights and had higher prevalence of obesity and glucose intolerance in adulthood (5).

In addition to under-nutrition, over-nutrition can also impact the metabolic health of the offspring (2). The relationship between fetal overnutrition and maternal obesity had been shown to also increase the incidence of large-for gestational age, offspring obesity, and risk of long-term morbidity (6–9). Previous studies of gestational over-nutrition in non-human primate revealed that offspring are more predisposed to metabolic diseases later in life due to changes in islet composition, glucose response, and insulin secretion (10). In a preclinical model in the rat, maternal high-fat diet throughout gestation and lactation promotes the onset of diabetes (11). The 1^st^ (F1) and 2^nd^ (F2) generation adult offspring of dams fed high-fat diet (8 weeks before pregnancy until end of lactation period) show impaired glucose metabolism and beta-cell dysfunction (12). In mice, prolonged prepregnant maternal high-fat diet (12 weeks) was sufficient to enhance beta-cell mass in the offspring (13). Maternal high-fat diet before pregnancy and during gestation and lactation were also shown to impair metabolic health associated with increased islet sizes (14). These changes can affect the pancreas, leading to metabolic dysfunction and ultimately, T2D (15).

There is, however, little known regarding the impact of maternal high-fat diet exposure shorty before and only during pregnancy on the pancreatic beta-cell and alpha cell ratios in neonatal offspring (F1). Therefore, we aimed to assess the impact of maternal high-fat diet four-weeks before and during gestation on the glucose metabolism and beta-cell function in early life and adult offspring in mice. To this end, we generated an animal model of maternal obesity and characterized glucose homeostasis and beta-cell mass. Here, we report that offspring of HFD-fed dams show reduced pancreatic beta-cell ratio at birth. Adult female offspring under normal diet display insulin resistance associated with increased insulin secretion. Male adult offspring of HFD-fed dams do not show any metabolic dysfunction under normal chow diet or in high-fat diet re-challenge compared to control groups. These data suggest that female offspring are negatively impacted by maternal high-fat diet programming.

## Results

### Reduced Pancreatic Beta-Cell Fraction in Newborns of Dams Fed High-Fat Diet

**Figure 1:**
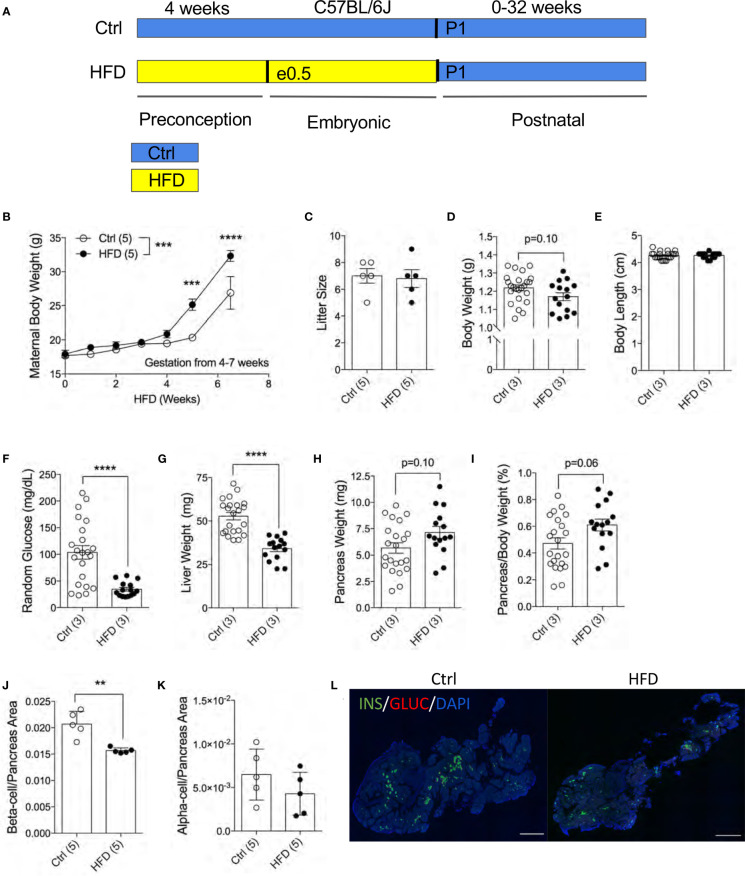
Reduced pancreatic beta-cell fraction in F1 newborns of dams fed high-fat diet. Experimental design schematic of obese dams and HFD during gestation **(A)**. Maternal body weight measured under HFD treatment 4 weeks prior and 3.5 weeks during pregnancy (n=5 dams per treatment, **B**). Litter size measured at P7 of Ctrl versus HFD dams, (n=5 dams per treatment, **C**). Body weight in offspring at P1 (n=24, 15 Ctrl and HFD offspring, **D**). Body length of offspring at P1 measured from rump to tail (n=22, 15 Ctrl and HFD offspring, **E)**. Random blood glucose measured from trunk blood of P1 offspring (n=22, 15 Ctrl and HFD offspring, **F**). Liver weight of P1 offspring (n=22, 15 Ctrl and HFD offspring, **G**). Pancreas weight and adjusted pancreatic weight to body weight of P1 offspring (n=22, 15 Ctrl and HFD offspring **H, I**). Beta-cell and alpha-cell ratio of P1 offspring (n=5 Ctrl and HFD offspring, **J, K**). Representative IF pancreatic staining at 10x on P1 Ctrl and HFD offspring **(L)**. At least 3 litters per treatment were used in **(D–K)**. Statistical analysis was performed using two-way ANOVA Sidak’s multiple comparisons **(B)** and Mann-Whitney two-tailed t-test **(C–K)**. Treatment effect variance was significant in (symbolized as *, **B**) with no interaction effect. Error bars represented ± SEM. **p < 0.01, ***p < 0.001, ****p < 0.0001 Ctrl vs. HFD. Scale bars are 500 µm.

To understand the effects of maternal HFD programming in the F1 offspring, we generated an experimental model with HFD treatment four weeks before and during pregnancy (**Figure 1A**). To assess the effects of HFD on the dams, we measured maternal body weight weekly during HFD treatment. Both Ctrl and HFD-dams displayed increased weight during pregnancy (**Figure 1B**). A significant difference in weight gain was observed between HFD-dams and Ctrl-dams at 4 and 7 weeks during gestation (**Figure 1B**). No significant changes in maternal insulin or glucagon levels between groups (data not shown). Litter size at birth (P0) was comparable between HFD and Ctrl dams (**Figure 1C**). The body weight at postnatal day 1 (P1) in HFD offspring was normal compared to Ctrl offspring (p=0.10, **Figure 1D**). We measured body length from rump to tail and found no differences between the groups (**Figure 1E**). Random blood glucose levels were significantly reduced in HFD offspring compared to Ctrl (**Figure 1F**). Liver weight was significantly reduced in HFD offspring compared to Ctrl (**Figure 1G**). No change in pancreatic weight was found between the F1 groups (p=0.10, **Figure 1H**), however, when adjusted to body weight, non-significance change was seen in HFD compared to Ctrl offspring (p=0.06, **Figure 1I**). Pancreatic beta-cell ratio at P1 was significantly decreased in HFD offspring compared to Ctrl (**Figure 1J**), however, alpha-cell ratio was comparable between the groups (**Figures 1K, L** and **Supplemental Figure 1A**). Together, these data suggest that maternal HFD exposures *in utero* promotes reduced liver weight and beta-cell ratio in neonates.

### Adult Male Offspring of HFD Dams Are Metabolically Normal Under Normal Chow Diet and Display a Transient Glucose Intolerance in HFD Challenge

**Figure 2:**
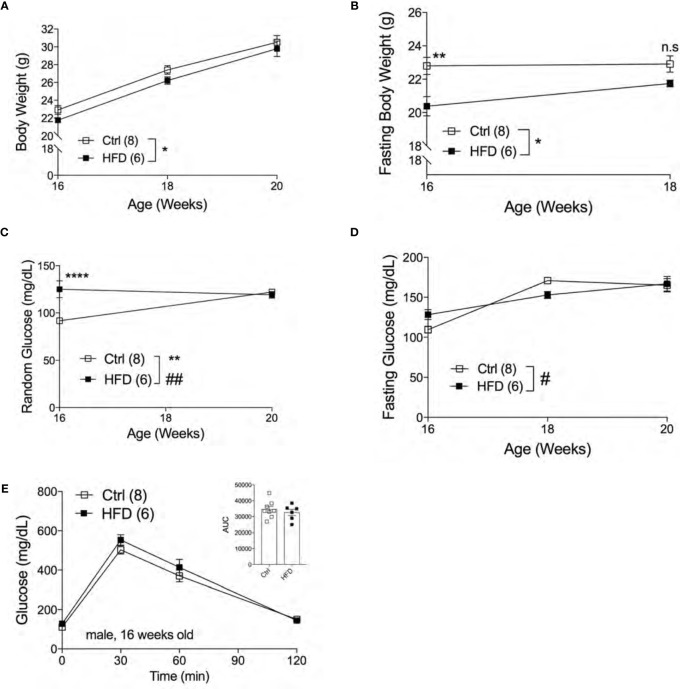
Adult male offspring of HFD dams are metabolically normal under normal chow diet. Body weight of males measured from 16 weeks to 20 weeks of age (n=8, 6 Ctrl and HFD offspring, **A**) and fasting body weight from 16 to 18 weeks old **(B)** under Ctrl diet. Random and fasting blood glucose of 16 to 20 weeks old males (n=8, 6 Ctrl and HFD offspring, **C, D**) under Ctrl diet. Intraperitoneal glucose tolerance test (IPGTT) of 16 week-old males with AUC (n=8, 6 Ctrl and HFD offspring, **E**) under Ctrl diet. At least 3 litters per treatment used in **(A–E)**. Statistical analysis was performed using two-way ANOVA Sidak’s multiple comparisons **(A–E)**. Error bars represented ± SEM. *p < 0.05, **p < 0.01, ****p < 0.0001 Ctrl vs. HFD. ^#^p < 0.05, ^##^p < 0.01 Ctrl vs. HFD. Interaction and treatment effects indicated as # and *, respectively.

**Figure 3:**
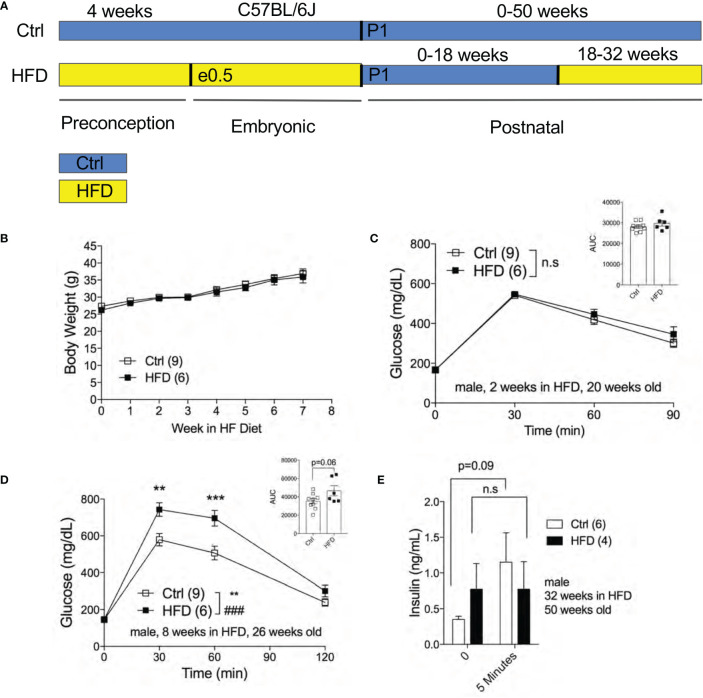
Adult male offspring of HFD and Ctrl dams show comparable glucose homeostasis in HFD challenge. Experimental flow chart for male offspring of Ctrl and HFD dams **(A)**. Body weight of males in HFD over 7 weeks starting at 18 weeks old (n=9, 6 Ctrl and HFD offspring, **B**). IPGTT of males at 2 and 8 weeks of HFD (n=9, 6 Ctrl and HFD offspring, **C, D**) with AUC at 20 and 26 weeks old. *In vivo* GSIS of males at 32 weeks of HFD at 50 weeks old (n=6, 4 Ctrl and HFD offspring, **E**). Statistical analysis was performed using two-way ANOVA Sidak’s multiple comparisons **(B–E)**. At least 3 litters per treatment used in **(B–E)**. Error bars represented ± SEM. **p < 0.01, ***p < 0.001, Ctrl vs. HFD. ^###^p < 0.001 Ctrl vs. HFD. Interaction and treatment effects indicated as # and *, respectively. Non-significance indicated as n.s.

The effect of fetal programming is often sex specific. To assess sex dimorphism effects of maternal HFD programming, we followed separate cohorts of adult male and female offspring and performed metabolic phenotyping tests. The body weight of adult male offspring was unchanged from 16-20 weeks of age in Ctrl within respective group (**Figure 2A**). When fasted, HFD male offspring have significantly lower body weight at 16 weeks of age but not at 18 weeks (**Figure 2B**). Random (non-fasted) blood glucose was significant at 16 weeks for HFD offspring (**Figure 2C**). Fasted blood glucose levels were comparable between male offspring of HFD and Ctrl groups (**Figure 2D**). The intraperitoneal glucose tolerance test (IPGTT) was comparable between male offspring from Ctrl and HFD dams (**Figure 2E**). Since there were no differences in glucose homeostasis phenotypes in male offspring under Ctrl, we introduced a “second hit” challenge, which is a diet-induced obesity by HFD rechallenge. See **Figure 3A** for experimental scheme. We monitored body weight of adult male offspring from Ctrl and HFD dams, and we detected no differences post 7 weeks of HFD diet treatment (**Figure 3B**). Glucose response in IPGTT testing did not show any differences between the two groups at 2 weeks of HFD treatment (**Figure 3C**). Albeit a trend toward increased glucose intolerance (AUC, p=0.06) was observed in male HFD offspring after 8 weeks of HFD (**Figure 3D**), no differences in glucose tolerance was evident between the groups after 12 and 32 weeks of HFD (**Supplemental Figures 2A, B** **)**. *In vivo* glucose-stimulated insulin secretion (GSIS) showed a non-significant differences at either time point 0 and 5 minutes between Ctrl and HFD offspring treated with HFD (**Figure 3E**). However, the Ctrl animals secreted insulin in response to glucose (p=0.09), whereas the HFD offspring were nonresponsive to glucose (**Figure 3E**) after 32-weeks of HFD. These data show that adult male offspring of HFD dams display comparable glucose tolerance as age-matched control (offspring of Ctrl dams) under Ctrl and HFD diets.

### Adult Female Offspring of HFD Dams Display Insulin Resistance in Normal Chow Diet

**Figure 4:**
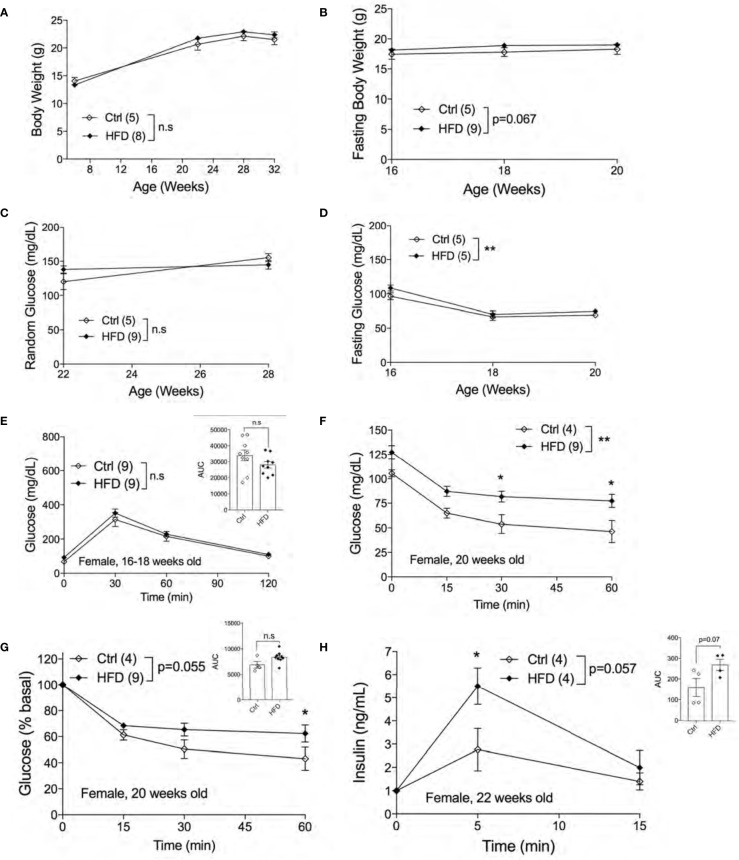
Adult female offspring of HFD dams display insulin resistance in normal chow diet. Body weight of females measured from 7-32 weeks of age (n=5, 8 ctrl and HFD offspring, **A**). Fasting body weight measured from 16-20 weeks of age (n=5, 9 Ctrl and HFD offspring, **B**). Random blood glucose of females measured at 22 and 28 weeks (n=5, 9 Ctrl and HFD offspring, **C**). Fasting blood glucose measured from 16-20 weeks of age (n=5 Ctrl and HFD offspring, **D**). IPGTT of females at 16-18 weeks old and AUC insert (n=9 Ctrl and HFD offspring, **E**). ITT of 20-week-old female and adjusted by % basal of glucose with AUC insert (n=4, 9 Ctrl and HFD offspring, **F, G**). *In vivo* GSIS of females at 22 weeks old (n=4 Ctrl and HFD offspring, **H**) and AUC insert. At least 3 litters per treatment were used **(A–H)**. Statistical analysis was performed using two-way ANOVA Sidak’s multiple comparisons **(A–H)**. No significant interaction effect was discovered. Significant treatment effects were indicated as *. Error bars represented ± SEM. *p < 0.05, **p < 0.01 Ctrl vs. HFD. Non-significance indicated as n.s.

Next, we assessed the metabolic phenotypes of female offspring of HFD-fed and Ctrl dams in Ctrl. No differences in random body weight were detected from ages 7 weeks to 32 weeks between adult female offspring of HFD and Ctrl dams (**Figure 4A**) or in fasting body weights (**Figure 4B**). Both non-fasted and fasted blood glucose levels were unchanged between groups (**Figures 4C, D** **)**. Comparable glucose tolerance was observed in 16-18 weeks old female offspring of HFD and Ctrl dams (**Figure 4E**). We then performed ITT in 20 weeks old females to test for insulin sensitivity in the peripheral tissues. Interestingly, we found insulin resistance in female offspring of HFD compared to offspring of Ctrl dams, figures are shown in raw and % basal values, respectively (**Figures 4F, G** **)**. Hyperinsulinemia is often associated with insulin resistance. To check whether the insulin resistance phenotype was accompanied by insulin secretion defects, we performed an *in vivo* GSIS experiment. *in vivo* GSIS was significantly increased in female offspring of HFD vs. Ctrl dams at 22 weeks (**Figure 4H**). These data suggest that the pancreatic beta-cells were responding to the insulin resistance present in the female offspring of HFD dams.

### Adult Female Offspring of HFD Dams Show Normal Beta-Cell Mass in Normal Chow Diet

**Figure 5:**
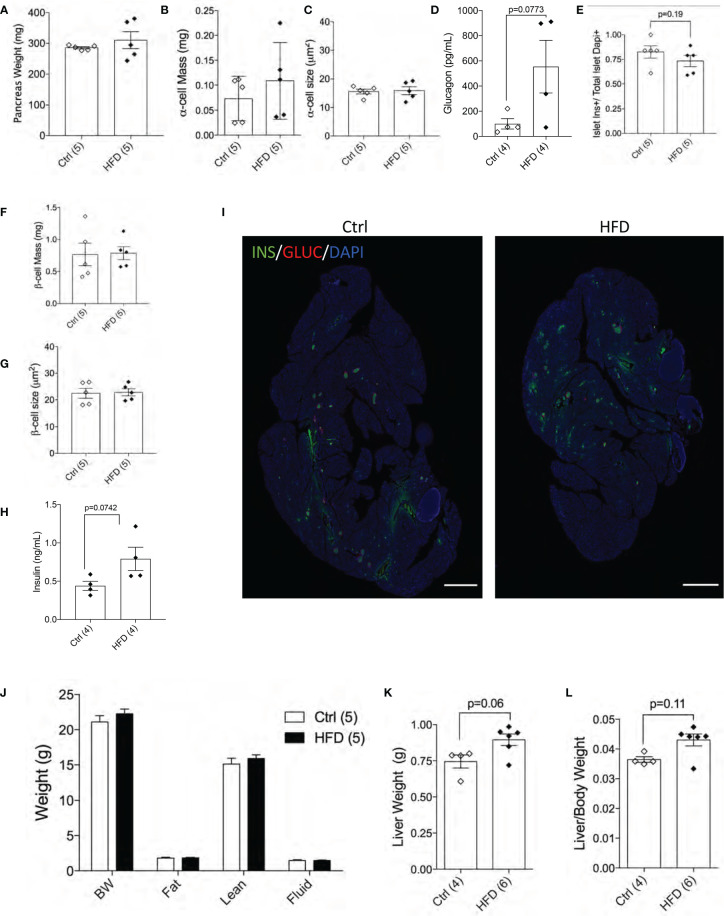
Female offspring of HFD dams show normal beta-cell mass in normal chow diet. Pancreas weight of Ctrl versus HFD females, α-cell mass, α-cell size (n=5 Ctrl and HFD offspring, **A–C**) and plasma glucagon (n=4 Ctrl and HFD offspring, **D**). Islet composition proportion for insulin-positive cells (n=5 Ctrl and HFD offspring, **E**). Average β-cell mass and β-cell size at 42 weeks in Ctrl diet (n=5 Ctrl and HFD offspring, **F, G**), and plasma insulin (n=4 Ctrl and HFD offspring, **H**). Representative IF pancreatic images at 10x of Ctrl and HFD adult offspring at 42 weeks **(I)**. Body composition measured on 32-week-old females in Ctrl versus HFD (n=5 Ctrl and HFD offspring, **J**). Liver weight of females and adjusted by body weight (n=4, 6 Ctrl and HFD offspring, **K, L**). At least 3 litters per treatment were used **(A–J)**. Statistical analysis was performed using two-way ANOVA Sidak’s multiple comparisons **(J)** and Mann-Whitney two-tailed t-test **(A–H, K, L)**. No significant interaction effect was found. Error bars represented ± SEM. Scale bars are 500 µm.

Next, we performed morphological analysis and measured pancreatic beta-cell mass in both groups. No differences in total pancreatic mass, alpha-cell mass, alpha-cell size and glucagon levels were detected between female offspring of HFD and Ctrl dams (**Figures 5A–D**). Beta-cell number per islet (p=0.19) and beta-cell mass were similar among the groups tested (**Figures 5E, F** **)**. Beta-cell size and plasma insulin were the same between the two groups (**Figures 5G, H** **)** with representative images shown of the pancreas from Ctrl and HFD offspring (**Figure 5I** and **Supplemental Figure 3A**). When body composition at 32 weeks was performed in adult female offspring of HFD and Ctrl dams, no changes in body weight, fat, lean or fluid mass were detected between the two groups (**Figure 5J**). Liver weight (adjusted to body weight) comparison showed non-significance in female offspring of HFD dams (p=0.06, p=0.11, **Figures 5K, L** **)**. These data show that adult female offspring of HFD dams develop insulin resistance associated with increased insulin secretion.

## Discussion

In the present study, we characterized the metabolic phenotypes of the newborn and adult female and male offspring of dams fed HFD during pre-conception (4 weeks) and throughout pregnancy. Our results reveal that neonates of HFD-fed dams have reduced beta-cell ratio associated with reduced non-fasted glucose levels. In adulthood, male and female offspring display normal glucose tolerance. However, female offspring of HFD-fed dams, but not male offspring, display insulin resistance and normal beta-cell mass phenotypes under normal diet. When adult male offspring of HFD-dams were re-challenged to a HFD model of diet-induced obesity, they showed comparable responses (weight gain, glucose, and insulin tolerance) to offspring of Ctrl-diet fed dams. These data suggest that female offspring were significantly impacted by maternal high-fat diet during gestation, however, the mechanism remains unknown.

Several studies have shown that maternal obesity alters the beta-cell mass in the offspring. One of our aims was to investigate the effects of a maternal HFD on beta-cell and alpha cells in the newborn offspring. In the current study we have found that at birth, murine offspring of maternal obesity have reduced beta-cell mass. Cerf et al. also reported reduced beta-cell volume and numbers in offspring in the rat of dams fed HFD during pregnancy (16). A reduction in beta-cell ratio was also reported in maternal low-protein diet during pregnancy in the offspring, where a reduced beta-cell mass was evident in newborn pancreas (3). We observed normal alpha cell ratio in the newborn and adult offspring of HFD-dams. However, in Japanese macaques, longer exposure to western-style diet *in utero* and throughout the lactation period reduces alpha cell mass in the offspring at three years of age (10).

The effect of maternal obesity on adult offspring beta-cell mass has been investigated but the results are conflicting even if the duration period (i.e., pre-conception and throughout pregnancy +/- lactation period) of the HFD treatment is the same, and the effect is often sex specific. Elzakr et al. wrote a comprehensive review on this topic (2). For most investigations into the effects of maternal obesity and multi-parity (a risk factor for maternal obesity) there is a worsened metabolic outcome for the offspring (2, 17). In our study, although both adult male and female offspring under normal chow diet displayed normal glucose tolerance, the male offspring re-challenged with HFD developed glucose intolerance. In our study, we also uncovered that female offspring developed insulin resistance under normal chow diet. Using a similar experimental design, Chang et al. observed that the adult male offspring of maternal obesity exhibited a mild glucose intolerance, whereas female offspring displayed normal glucose tolerance (18). However, in this study by Chang et al., insulin tolerance was not tested in the female offspring (18). In our study, however, the female offspring displayed insulin resistance associated with increased insulin secretion but normal beta-cell mass. Because the insulin tolerance test is a crude assessment of insulin sensitivity, additional testing using the gold standard hyperinsulinemic/euglycemic clamp is needed to confirm insulin resistance in the offspring. In both human and animal studies, insulin resistance is associated with hyperinsulinemia. Thus, an increased in insulin secretion was expected as observed phenotype in the offspring of obese dams. Interestingly, Nicholas et al. also observed improved islet function, where female islets but not male offspring appear to be programmed to cope with a nutritionally rich postnatal environment by increasing insulin secretion, mitochondrial respiration, and beta-cell survival (19). In Agouti mutation model that produces marked obesity without diabetes in the dam, adult female offspring show glucose intolerance associated with insulin secretion defect in beta-cells (20). It is important to point out that other studies report that male offspring display glucose intolerance with impaired insulin secretion. However, they demonstrated a compensatory increase in pancreatic beta-cell mass and a decreased in glucose-stimulated insulin secretion from isolated islets (14, 21). The effects of maternal programming in beta-cell can continue into the 2^nd^ generation as well: Huang et al, uncovered reduced beta-cell mass in 3- or 12-week-old F2 rats (12). It is clear that maternal obesity and overnutrition have been shown to impact beta-cell mass and function in rodents and ewes (22–24) despite their capability to adapt or compensate in the changes of the intrauterine environment over time (25). The mechanisms by which beta-cell number and function are altered in the offspring of dams fed high-fat diet are elusive, but placental nutrient flux (e.g. amino acids, glucose, lipids) to the fetus could play a role given that beta-cells are very sensitive to nutrient levels early in life (3, 26). Thus, an unbiased transcriptome and proteome studies on adult offspring islets can give insight on the potential key players modulating the effects of maternal obesity. In addition, the mechanism(s) by which insulin resistance is programmed or which tissue(s) impacted by maternal obesity remain unknown. In the current study, we observed a reduction in liver weight in neonates and increased in adulthood, and the metabolic health consequence(s) of this phenotype should be investigated in the future. Epigenetic modifications can play a role in the programming of insulin resistance by maternal programming (27–30), and this should be pursued mechanistically in multiple tissues including the liver.

There are several limitations in the current study that would serve useful in deepening our understanding of the susceptibility, metabolic profile, and sex dimorphism effect of the offspring.

Assessment of beta-cell function in newborns and segregation of data by sex would have been more informative. Future research directions of gestational fetal programming should investigate the molecular mechanisms of sex specific outcomes. Differences in placental gene expression and inflammation response, epigenetic mechanisms in fetal gene expression (i.e., *Insulin 1 and Insulin 2* DNA methylation), and hormone levels could all play a role in fetal programming by maternal obesity that we intend to further investigate. An interesting idea that has not been studied, is the possible increased risk of type 1 diabetes in the offspring of obese dams. It is important to understand how children of obese mothers are predisposed to exacerbated obesity and diabetes so we can prevent and lessen the viscous cycle of diabetes.

## Materials and Methods

### Animal Generation and Experimental Design

Naïve C57BL/6J mice were used and purchased from The Jackson Lab. See **Figure 1A** for experimental design graphic. At least 5 dams (or litters) per treatment were used to generate the experimental litters. One litter is considered n of 1. The offspring from at least 3 litters per treatment were used. The two dam groups were then set up for time pregnancies in the evenings and a detection of a vaginal plug indicates embryonic day 0.5 (e0.5). Dams were either given normal chow diet (NCD) 10% kcal fat (referred to as Ctrl, D12450B, Research Diets) or high-fat diet 60% kcal fat (referred to as HFD, D12492, Research Diets) 4 weeks before and during pregnancy ad libitum. At birth, the offspring was categorized based on the treatment diet of the dam during gestation: F1 Ctrl or HFD offspring are from Ctrl or HFD dams, respectively. The dams were then placed on a Ctrl diet during the lactation period followed by offspring weaning on Ctrl diet. Male and female cohorts were followed and assessed in the current study from birth to 32 weeks postnatal. Male cohorts were additionally tested up to 32 weeks of HFD, or up to 50 weeks of age. See **Figure 3A** for male HFD experimental flow chart. A separate female cohort was generated to assess pancreatic beta-cell mass in female offspring. The same paradigm as in **Figure 1A** except Ctrl is 13% kcal fat (PicoLab Rodent Diet 5L0D) (18). All studies were approved by the Institutional Animal Care and Use Committee at the University of Michigan.

### Tissue Collection

Pancreatic tissues were formalin-fixed and embedded in paraffin using standard procedures as previously described (4). Neonatal pancreata (P1) were harvested at birth and fixed in 3.7% formalin for 6 hours prior to 70% ethanol treatment and processing. Female adult pancreas was collected at 6 months of age and fixed in 3.7% formalin for 12 hours, following 70% ethanol and processing.

### Beta and Alpha-Cell Ratio and Beta-Cell Mass Analysis in Newborns

Newborn and adult beta-cell and alpha-cell ratio was performed using staining and analysis procedures as previously described (4, 31) using insulin (Sigma, I8510) and glucagon (Abcam, ab92517) antibodies. Adult female offspring samples were from treatment groups Ctrl 13% and HFD for beta-cell ratio. Newborn pancreatic tissues were sectioned thoroughly at 5 μm, and adult pancreas was collected in five regions, where one region is 50 μm total sectioned at 5 μm each. Each new region was reached after shaving off an additional 200 μm. Beta-cell size was measured by taking the ratio of beta-cell area over count over number of beta-cells. All image analysis and quantification were done using ImageJ (NIH, http://imagej.nih.gov/ij/).

### Glucose Homeostasis Studies and Insulin/Glucagon ELISA

Metabolic studies and *in vitro* assays were performed as described (3). Intraperitoneal glucose tolerance test (IPGTT) was performed on mice after 15 hours of fasting with a dose of 2g/kg 50% dextrose solution (Hospira, RL-3040). Following intraperitoneal (IP) injection, glucose was measured at time points 0, 30, 60, and 120 from the tail vein. Insulin tolerance test (ITT) was performed on mice after 6 hours of fasting, following an IP injection of 0.75U/kg dose of insulin (Novolin, Novo Nordisk Inc.) in 0.9% saline. Sera was collected and stored in -80C until assessed for insulin or glucagon concentration with an ultrasensitive insulin or glucagon ELISA kit respectively (ALPCO). The results were analyzed on a 5-parameter logistics curve on MyAssayss software. Glucose stimulated insulin secretion (GSIS) was done after fasting of mice for 6 hours. An IP injection of 2g/kg glucose was performed following tail vein blood collection at time points 0, 3, and 5 minutes.

### NMR Analysis of Body Composition

NMR (Minispec LF90_II_, Bruker Biosciences Corp., Billerica, MA, USA) was used to investigate body composition through the University of Michigan Nutrition Obesity Research Center Animal Phenotyping Core.

### Statistical Analysis

Data shown is represented by mean +/- SEM. Statistical analysis was performed using two-tailed Mann-whitey t-test or two-way ANOVA adjusted for Sidak’s multiple comparisons as indicated in figure legends. P-value < 0.5 was considered significant. Data analysis was evaluated on GraphPad Prism 7 or 8 (GraphPad Software, San Diego, CA).

### Study Approval

All studies were approved by the Institutional Animal Care and Use Committee at the University of Michigan.

## Data Availability Statement

The original contributions presented in the study are included in the article/**Supplementary Material**. Further inquiries can be directed to the corresponding authors.

## Ethics Statement

The animal study was reviewed and approved by Institutional Animal Care & Use Committee University of Michigan.

## Author Contributions

EA and BA designed experiments, generated and analyzed data, interpreted the data, and wrote and edited the final manuscript. BG, SJ, DK, JS, and JD designed experiments and edited manuscript. KS and CL contributed to the discussion. EB-M edited the manuscript. All authors contributed to the article and approved the submitted version.

## Conflict of Interest

The authors declare that the research was conducted in the absence of any commercial or financial relationships that could be construed as a potential conflict of interest.

## Publisher’s Note

All claims expressed in this article are solely those of the authors and do not necessarily represent those of their affiliated organizations, or those of the publisher, the editors and the reviewers. Any product that may be evaluated in this article, or claim that may be made by its manufacturer, is not guaranteed or endorsed by the publisher.

## Supplementary Material

Supplementary Figure 1 Newborn Ctrl and HFD offspring pancreas image. Enlarged P1 newborn Ctrl and HFD offspring pancreas image of at 10x magnification. Insulin (green) staining localizes to beta-cells, glucagon (red) localizes to alpha-cells, and DAPI (blue) stains nuclei of the cells. Picture insets 1-4 are enlarged to show islet morphology at 10x. Scale bars are 500 µm.

Supplementary Figure 2 Male Ctrl and HFD offspring under HFD metabolic tests. IPGTT of males under 12 and 32 weeks of HFD with AUC **(**n=6, 5-6 Ctrl and HFD offspring, **A, B)**. At least 3 litters per treatment were used **(A, B)**. Error bars represented ± SEM. No significant interaction effect was found. Statistical analysis was performed using two-way ANOVA Sidak’s multiple comparisons **(A, B)**.

Supplementary Figure 3 Adult female Ctrl and HFD offspring pancreas image. Enlarged adult female Ctrl and HFD offspring pancreas image of at 10x magnification. Insulin (green) staining localizes to beta-cells, glucagon (red) localizes to alpha-cells, and DAPI (blue) stains nuclei of the cells. Picture insets 1-4 are enlarged to show islet morphology at 10x. Scale bars are 500 µm.

## References

[1] De Boo HA Harding JE. The Developmental Origins of Adult Disease (Barker) Hypothesis. Aust New Z J Obstet Gynaecol (2006) 46(1):4–14. doi: 10.1111/j.1479-828X.2006.00506.x 16441686

[2] Elsakr JM Gannon M. Developmental Programming of the Pancreatic Islet by *In Utero* Overnutrition. Trends Dev Biol (2017) 10:79–95. 29657386 PMC5894880

[3] Alejandro EU Gregg B Wallen T Kumusoglu D Meister D Chen A. Maternal Diet-Induced microRNAs and mTOR Underlie β Cell Dysfunction in Offspring. J Clin Invest (2014) 124(10):4395–410. doi: 10.1172/JCI74237 PMC4191023 25180600

[4] Alejandro EU Jo S Akhaphong B Llacer PR Gianchandani M Gregg B. Maternal Low-Protein Diet on the Last Week of Pregnancy Contributes to Insulin Resistance and β-Cell Dysfunction in the Mouse Offspring. Am J Physiol Regul Integr Comp Physiol (2020) 319(4):R485–96. doi: 10.1152/ajpregu.00284.2019 PMC7717124 32877242

[5] Roseboom T de Rooij S Painter R. The Dutch Famine and Its Long-Term Consequences for Adult Health. Early Hum Dev (2006) 82(8):485–91. doi: 10.1016/j.earlhumdev.2006.07.001 16876341

[6] Lawlor DA Smith GD O’Callaghan M Alati R Mamun AA Williams GM. Epidemiologic Evidence for the Fetal Overnutrition Hypothesis: Findings From the Mater-University Study of Pregnancy and Its Outcomes. Am J Epidemiol (2007) 165(4):418–24. doi: 10.1093/aje/kwk030 17158475

[7] Lawlor DA Timpson NJ Harbord RM Leary S Ness A McCarthy MI. Exploring the Developmental Overnutrition Hypothesis Using Parental-Offspring Associations and FTO as an Instrumental Variable. PloS Med (2008) 5(3):e33. doi: 10.1371/journal.pmed.0050033 18336062 PMC2265763

[8] Gaudet L Ferraro ZM Wen SW Walker M. Maternal Obesity and Occurrence of Fetal Macrosomia: A Systematic Review and Meta-Analysis. BioMed Res Int (2014) 2014:640291. doi: 10.1155/2014/640291 25544943 PMC4273542

[9] Boney CM Verma A Tucker R Vohr BR. Metabolic Syndrome in Childhood: Association With Birth Weight, Maternal Obesity, and Gestational Diabetes Mellitus. Pediatrics (2005) 115(3):e290–6. doi: 10.1542/peds.2004-1808 15741354

[10] Elsakr JM Dunn JC Tennant K Zhao SK Kroeten K Pasek RC. Maternal Western-Style Diet Affects Offspring Islet Composition and Function in a Non-Human Primate Model of Maternal Over-Nutrition. Mol Metab (2019) 25:73–82. doi: 10.1016/j.molmet.2019.03.010 31036449 PMC6599455

[11] Ohta T Toriniwa Y Ryumon N Inaba N Hirao T Yamanaka S. Maternal High-Fat Diet Promotes Onset of Diabetes in Rat Offspring. Anim Sci J (2017) 88(1):149–55. doi: 10.1111/asj.12606 27145882

[12] Huang YH Ye TT Liu CX Wang L Chen YW Dong Y. Maternal High-Fat Diet Impairs Glucose Metabolism, β-Cell Function and Proliferation in the Second Generation of Offspring Rats. Nutr Metab (Lond) (2017) 14:67. doi: 10.1186/s12986-017-0222-2 29118817 PMC5667458

[13] Qiao L Wattez JS Lim L Rozance PJ Hay WW Jr. Shao J. Prolonged Prepregnant Maternal High-Fat Feeding Reduces Fetal and Neonatal Blood Glucose Concentrations by Enhancing Fetal Beta-Cell Development in C57BL/6 Mice. Diabetes (2019) 68(8):1604–13. doi: 10.2337/db18-1308 PMC6692812 31127056

[14] Zheng J Zhang L Wang Z Zhang J. Maternal High-Fat Diet Regulates Glucose Metabolism and Pancreatic Beta Cell Phenotype in Mouse Offspring at Weaning. PeerJ (2020) 8:e9407. doi: 10.7717/peerj.9407 32607287 PMC7316079

[15] Mohan R Baumann D Alejandro EU. Fetal Undernutrition, Placental Insufficiency, and Pancreatic β-Cell Development Programming In Utero. Am J Physiol Regul Integr Comp Physiol (2018) 315(5):R867–78. doi: 10.1152/ajpregu.00072.2018 PMC6295492 30110175

[16] Cerf ME Williams K Nkomo XI Muller CJ Du Toit DF Louw J. Islet Cell Response in the Neonatal Rat After Exposure to a High-Fat Diet During Pregnancy. Am J Physiol Regul Integr Comp Physiol (2005) 288(5):R1122–8. doi: 10.1152/ajpregu.00335.2004 15705804

[17] Rebholz SL Jones T Burke KT Jaeschke A Tso P D’Alessio DA. Multiparity Leads to Obesity and Inflammation in Mothers and Obesity in Male Offspring. Am J Physiol Endocrinol Metab (2012) 302(4):E449–57. doi: 10.1152/ajpendo.00487.2011 PMC3287352 22127227

[18] Chang E Hafner H Varghese M Griffin C Clemente J Islam M. Programming Effects of Maternal and Gestational Obesity on Offspring Metabolism and Metabolic Inflammation. Sci Rep (2019) 9(1):16027. doi: 10.1038/s41598-019-52583-x 31690792 PMC6831633

[19] Nicholas LM Nagao M Kusinski LC Fernandez-Twinn DS Eliasson L Ozanne SE. Exposure to Maternal Obesity Programs Sex Differences in Pancreatic Islets of the Offspring in Mice. Diabetologia (2020) 63(2):324–37. doi: 10.1007/s00125-019-05037-y PMC6946752 31773193

[20] Han J Xu J Epstein PN Liu YQ. Long-Term Effect of Maternal Obesity on Pancreatic Beta Cells of Offspring: Reduced Beta Cell Adaptation to High Glucose and High-Fat Diet Challenges in Adult Female Mouse Offspring. Diabetologia (2005) 48(9):1810–8. doi: 10.1007/s00125-005-1854-8 16010523

[21] Yokomizo H Inoguchi T Sonoda N Sakaki Y Maeda Y Inoue T. Maternal High-Fat Diet Induces Insulin Resistance and Deterioration of Pancreatic Beta-Cell Function in Adult Offspring With Sex Differences in Mice. Am J Physiol Endocrinol Metab (2014) 306(10):E1163–75. doi: 10.1152/ajpendo.00688.2013 24691028

[22] Ford SP Zhang L Zhu M Miller MM Smith DT Hess BW. Maternal Obesity Accelerates Fetal Pancreatic Beta-Cell But Not Alpha-Cell Development in Sheep: Prenatal Consequences. Am J Physiol Regul Integr Comp Physiol (2009) 297(3):R835–43. doi: 10.1152/ajpregu.00072.2009 PMC2739779 19605766

[23] Long NM George LA Uthlaut AB Smith DT Nijland MJ Nathanielsz PW. Maternal Obesity and Increased Nutrient Intake Before and During Gestation in the Ewe Results in Altered Growth, Adiposity, and Glucose Tolerance in Adult Offspring. J Anim Sci (2010) 88(11):3546–53. doi: 10.2527/jas.2010-3083 20622177

[24] Zhang L Long NM Hein SM Ma Y Nathanielsz PW Ford SP. Maternal Obesity in Ewes Results in Reduced Fetal Pancreatic β-Cell Numbers in Late Gestation and Decreased Circulating Insulin Concentration at Term. Domest Anim Endocrinol (2011) 40(1):30–9. doi: 10.1016/j.domaniend.2010.08.004 PMC3008620 20933362

[25] Alejandro EU Gregg B Blandino-Rosano M Cras-Méneur C Bernal-Mizrachi E. Natural History of β-Cell Adaptation and Failure in Type 2 Diabetes. Mol Aspects Med (2015) 42:19–41. doi: 10.1016/j.mam.2014.12.002 25542976 PMC4404183

[26] Qiao L Shetty SK Spitler KM Wattez JS Davies BSJ Shao J. Obesity Reduces Maternal Blood Triglyceride Concentrations by Reducing Angiopoietin-Like Protein 4 Expression in Mice. Diabetes (2020) 69(6):1100–9. doi: 10.2337/db19-1181 PMC7243287 32051149

[27] Neri C Edlow AG. Effects of Maternal Obesity on Fetal Programming: Molecular Approaches. Cold Spring Harb Perspect Med (2015) 6(2):a026591. doi: 10.1101/cshperspect.a026591 26337113 PMC4743074

[28] Ross MG Desai M. Developmental Programming of Offspring Obesity, Adipogenesis, and Appetite. Clin Obstet Gynecol (2013) 56(3):529–36. doi: 10.1097/GRF.0b013e318299c39d PMC4191824 23751877

[29] Ganu RS Harris RA Collins K Aagaard KM. Early Origins of Adult Disease: Approaches for Investigating the Programmable Epigenome in Humans, Nonhuman Primates, and Rodents. Ilar J (2012) 53(3-4):306–21. doi: 10.1093/ilar.53.3-4.306 PMC3747760 23744969

[30] Sookoian S Pirola CJ. Epigenetics of Insulin Resistance: An Emerging Field in Translational Medicine. Curr Diabetes Rep (2013) 13(2):229–37. doi: 10.1007/s11892-012-0361-9 23307192

[31] Akhaphong B Baumann DC Beetch M Lockridge AD Jo S Wong A. Placental mTOR Complex 1 Regulates Fetal Programming of Obesity and Insulin Resistance in Mice. JCI Insight (2021) 6(13):e149271. doi: 10.1172/jci.insight.149271 PMC8410096 34032632

