# Supplementary material for: Maternal High-Fat Diet During Pre-Conception and Gestation Predisposes Adult Female Offspring to Metabolic Dysfunction in Mice
Source: Front Endocrinol (Lausanne). 2022 Jan 17;12:780300. doi: 10.3389/fendo.2021.780300 (PMC8801938; doi:10.3389/fendo.2021.780300)
Supplement: Supplementary Figure 1 — Newborn Ctrl and HFD offspring pancreas image. Enlarged P1 newborn Ctrl and HFD offspring pancreas image of at 10x magnification. Insulin (green) staining localizes to beta-cells, glucagon (red) localizes to alpha-cells, and DAPI (blue) stains nuclei of the cells. Picture insets 1-4 are enlarged to show islet morphology at 10x. Scale bars are 500 µm. [file DataSheet_1.pdf]

Supplemental Figure 1

A

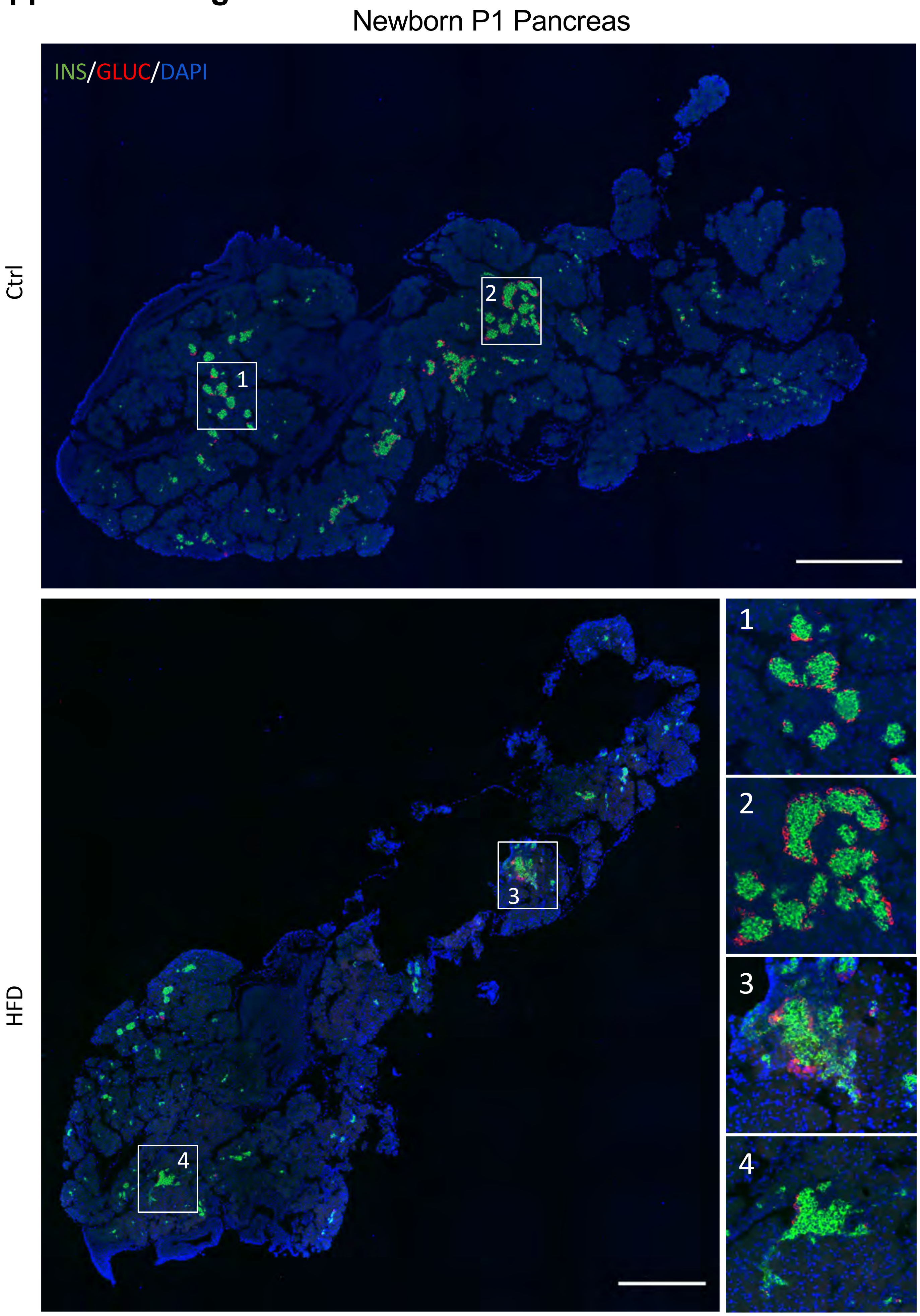

Supplemental Figure 2

A

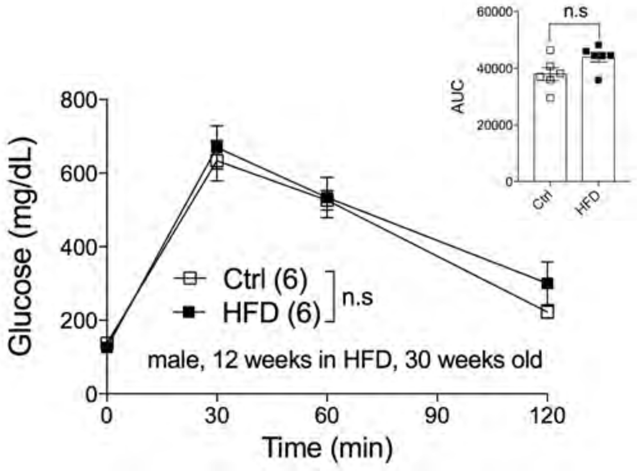

B

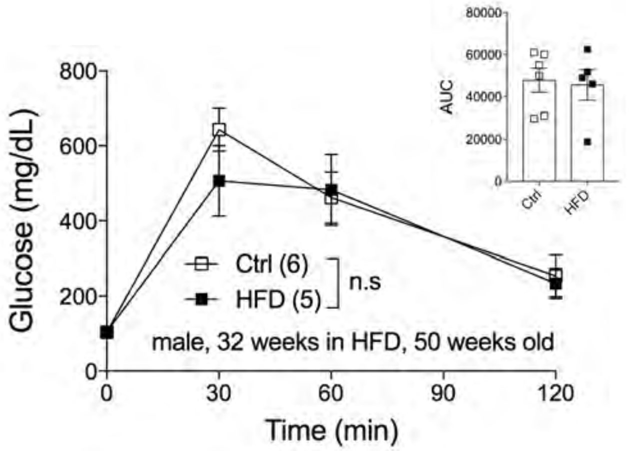

Supplemental Figure 3

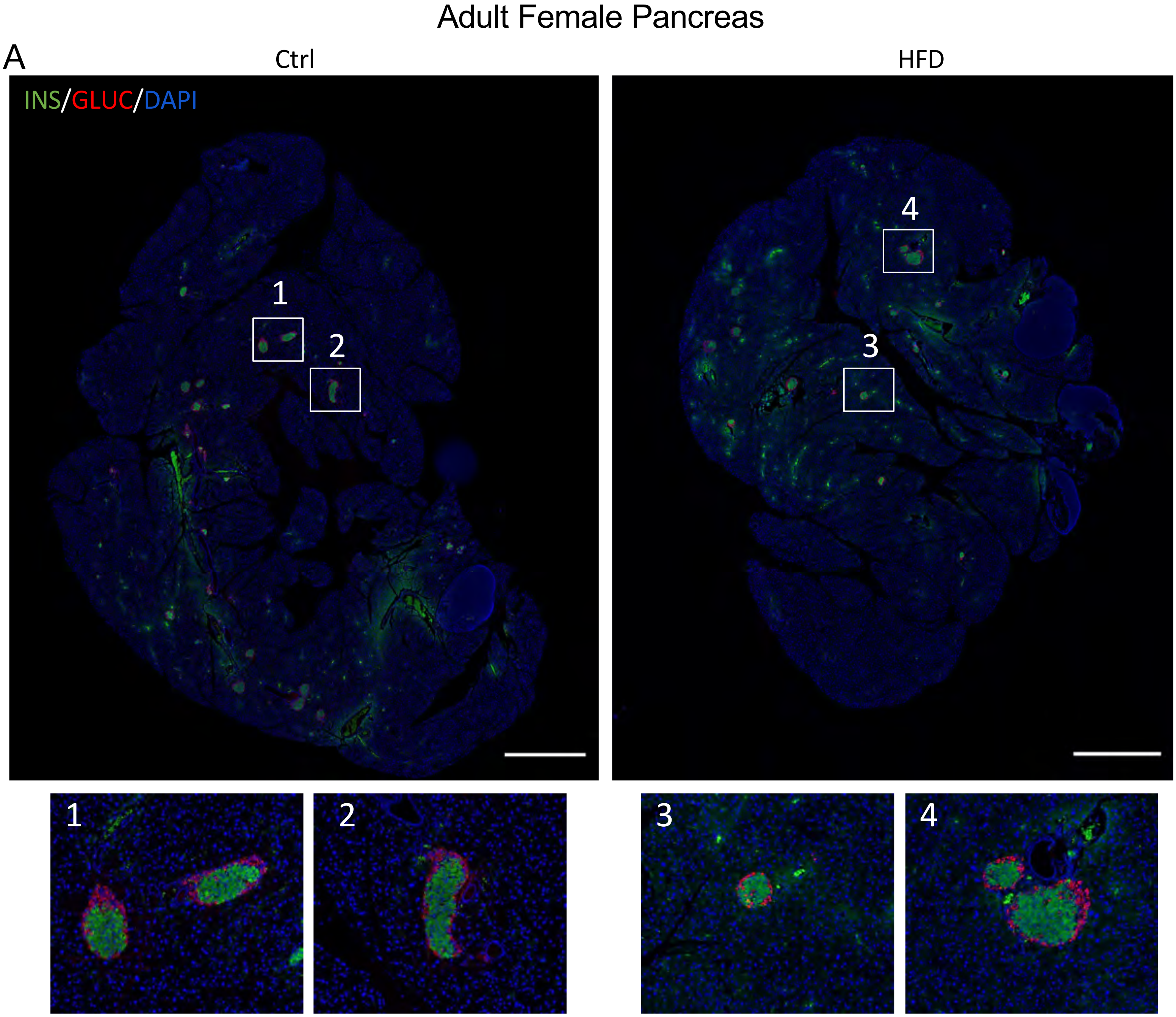

Legends

Supplemental Figure 1. *Newborn Ctrl and HFD offspring pancreas image*

Enlarged P1 newborn Ctrl and HFD offspring pancreas image of Figure 1L at 10x magnification. Insulin (green) staining localizes to beta-cells, glucagon (red) localizes to alpha-cells, and DAPI (blue) stains nuclei of the cells. Picture insets 1-4 are enlarged to show islet morphology at 10x. Scale bars are 500 μm.

Supplemental Figure 2. *Male Ctrl and HFD offspring under HFD metabolic tests*

IPGTT of males under 12 and 32 weeks of HFD with AUC (n=6, 5-6 Ctrl and HFD offspring, **A**, **B**). At least 3 litters per treatment were used (**A**, **B**). Error bars represented ± SEM. No significant interaction effect was found. Statistical analysis was performed using two-way ANOVA Sidak's multiple comparisons (**A**, **B**)

Supplemental Figure 3. *Adult female Ctrl and HFD offspring pancreas image*

Enlarged adult female Ctrl and HFD offspring pancreas image of Figure 5G at 10x magnification. Insulin (green) staining localizes to beta-cells, glucagon (red) localizes to alpha-cells, and DAPI (blue) stains nuclei of the cells. Picture insets 1-4 are enlarged to show islet morphology at 10x. Scale bars are 500 μm.
